# Retraction: Ectopic expression of ROR1 prevents cochlear hair cell loss in guinea pigs with noise‐induced hearing loss

**DOI:** 10.1111/jcmm.18169

**Published:** 2024-02-09

Jun Zhang, Wei Zhang, Qinliang Zhang, Ectopic expression of ROR1 prevents cochlear hair cell loss in guinea pigs with noise‐induced hearing loss. J Cell Mol Med. 2020; 24: 9101–9113. https://doi.org/10.1111/jcmm.15545.

The above article, published online on 07 July 2020 in Wiley Online Library (wileyonlinelibrary.com), has been retracted by agreement between the authors, the journal Editor‐in‐Chief, Stefan Constantinescu, The Foundation for Cellular and Molecular Medicine and John Wiley and Sons Ltd. The authors asked to retract the article as they found that the experiments presented were not reproducible; therefore, the conclusions of the paper are not supported. Although initially the corresponding author contacted the editorial office and asked for retraction, they did not respond when asked to approve the wording.

